# Bromodomain and Extra-Terminal (BET) Domain Protein Inhibitors for Solid Tumor Cancers

**DOI:** 10.4103/JIPO.JIPO_2_20

**Published:** 2020-02-05

**Authors:** Martin V. Nguyen, Lydia Loof, Gerald S. Falchook

**Affiliations:** 1 Sarah Cannon Research Institute at HealthONE, Denver, CO, USA

**Keywords:** Bromodomain and extraterminal domain, bromodomain and extraterminal inhibitor, cancer, clinical trial, review

## Abstract

The bromodomain and extraterminal (BET) domain protein family is involved in the process of transcription of genetic information. The BET protein family includes BRD2, BRD3, BRD4, and bromodomain testis-specific protein. BET protein alterations are associated with some solid tumor cancers, including nuclear protein in testis midline carcinoma. BET protein has a role in carcinogenesis and in the regulation of the cell cycle. A number of BET inhibitors have entered clinical trials. This review discusses the results of BET inhibitor clinical trials in solid tumor cancers.

## Introduction

The bromodomain and extraterminal (BET) domain protein family is involved in the process of transcription of genetic information.[1] The BET protein family includes BRD2, BRD3, BRD4, and bromodomain testis-specific protein.[1–3] Acetylation of histones via histone acetyltransferases removes the partial positive charge from the histone, leading to decreased interaction between the partial negative charge of the phosphate groups in DNA.[3] This event causes the DNA to transit from heterochromatin (highly condensed, transcriptionally inactive) to euchromatin (relaxed, transcriptionally active). BRD4 selectively binds to acetylated lysine residues on histones and serves as epigenetic readers, which recruits other subunits and proteins for DNA transcription. BRD4 functions as a mitotic bookmark for genes to be transcribed in early G1 phase.[4,5] BRD2 is suspected to interact with transcription factors which involve RNA polymerase II, resulting in the formation of a protein complex which initiates transcription.[5] BET proteins are also involved in kinase activity, but the specific function has not yet been identified.[3]

Because normal BET protein function involves transcription and cell proliferation, aberration of these proteins contributes to genetic mutations and uncontrolled cell proliferation. In nuclear protein in testis (NUT) midline carcinoma (NMC), BRD4 forms a fusion oncoprotein as a result of a translocation which drives the development of the disease.[4] BET proteins also play a role in carcinogenesis because of their role in the regulation of the cell cycle. BRD4 protein is related to c-MYC transcription.[1,2] C-MYC is a proto-oncogene that is involved in the proliferation and growth mechanisms of the cell. C-MYC translocation occurs in various hematologic malignancies, which can drive carcinogenesis.[1,3–5] In solid tumor cancers, c-MYC overexpression and amplification have been observed and are also implicated in carcinogenesis.[3]

BET inhibitors competitively bind to the same site similar to the acetyl lysine histones. This downregulates transcription expression and causes G1 arrest in the cell. However, BET inhibitors selectively bind to superenhancers within cancer cells and do not affect the surrounding wild-type cells.[5] BET inhibitors have been tested with in-vitro and in-vivo models and have shown that inhibition of BET proteins downregulates MYC expression.[5] When treated with a BET inhibitor JQ1, mice with MYC-amplified tumors showed more intense effects of cell apoptosis and inhibited cell differentiation.[3,4] However, in another preclinical testing of BET inhibitors, there was no correlation between MYC mRNA levels and drug exposure.[3,5] The relationship between MYC downregulation and BET inhibition is still under investigation.

Preclinical studies of first-generation synthetic BET inhibitors, such as JQ1 (thienotriazolodiazepine), have shown anticancer activity in murine and xenograft models of NMC, acute myeloid leukemia (AML), multiple myeloma, and Burkitt's lymphoma.[3] Specifically in NMC, suppression of the BRD4–NUT fusion gene that is known to drive NMC growth has resulted in the growth arrest of NMC cells. In AML, preclinical studies have found that BET inhibitors may be able to successfully block the expression of c-MYC.[6] Significant antiproliferative results were also presented in a study, in which five T-cell lymphoma cell lines were incubated with JQ1, which also resulted in decreased c-MYC levels in all cell lines except one.[7] While various studies have demonstrated the preclinical efficacy of BET inhibition as monotherapy, combination regimens may also be promising. BET inhibitors have demonstrated synergy with immune checkpoint inhibitors and epigenetic agents, specifically histone deacetylase inhibitors.[5]

Toxicities observed in the preclinical setting include depletion of hematopoietic cells, skin hyperplasia, and disruption of the intestinal crypts in mice with reduced BRD4 levels; neuronal defects and obesity in mice with reduced BRD2 levels; and impaired long-term memory and heightened anxiety in mice receiving JQ1.[3]

Early-stage clinical trials of BET inhibitors that target hematologic malignancies have demonstrated promising preliminary results. A Phase I, dose-finding trial of birabresib (previously MK-8628/OTX-015) (Merck Sharp and Dohme Corp., Kenilworth, NJ, USA) that started in December 2012 and ended in January 2017 observed antitumor responses in patients with leukemia and lymphoma. Among the 41 patients enrolled in the acute leukemia cohort of the study, three patients had a complete response (CR) (all the three with AML) and two had a partial response (PR) (one with AML and one with myelodysplastic syndrome). Among the 45 patients enrolled in the nonleukemic cohort of the study, two had a CR (both with diffuse large B-cell lymphoma [DLBCL]), one had a PR (with DLBCL), and six had stable disease (SD) (four with DLBCL and two with indolent lymphoma). While no patients discontinued treatment due to adverse events (AEs), the most common AEs in the acute leukemia cohort were gastrointestinal (vomiting, nausea, and diarrhea), fatigue, and hyperbilirubinemia. Similar AEs were observed in the nonleukemic cohort, but cytopenias were more prevalent (58% experienced Grade [G] 3 thrombocytopenia). Preliminary results from both trials concluded that birabresib can be safely administered in patients with advanced hematologic malignancies.[3,8–10]

Antitumor activity in hematologic malignancies has also been observed in a Phase I trial of CPI-0610 (Constellation Pharmaceuticals, Cambridge, MA, USA) in patients with lymphoma that started in September 2013 and ended in December 2017. Of the 64 patients enrolled in the study, two had a CR (both with DLBCL), three had a PR (two with DLBCL and one with follicular lymphoma fl), and five (all with DLBCL) had SD of > 6 months. Thrombocytopenia was the most common AE (45%), but it was reversible and not cumulative.[11,12]

A Phase I trial of GSK525762 (GlaxoSmithKline, Brentford, UK) on 46 participants with AML also showed antitumor activity. Three patients had PRs and two had CRs (one with incomplete count recovery and one with incomplete platelet recovery). The most common AEs were dysgeusia (37%), diarrhea (33%), and nausea (28%). Preliminary results concluded that GSK525762 is safe and effective in patients with AML.[13,14]

An open-label safety and tolerability study of INCB057643 (Incyte Corporation, Wilmington, DE, USA) that started in May 2016 and ended in January 2019 showed antitumor activity in patients with FL. Of the 16 participants enrolled in the trial, three had FL, of which one participant had a CR and two had prolonged SD The only dose-limiting toxicity (DLT) was increased international normalized ratio (INR) in one patient, and the trial concluded that INCB057643 is safe and effective in patients with FL.[15,16]

This review discusses the BET pathway, the biological rationale for BET inhibitors, and the clinical trials involving BET inhibitors in solid tumor cancers.

## Bromodomain and Extraterminal Inhibitor Clinical Trials in Solid Tumor Cancers

### BAY1238097

BAY1238097 (Bayer, Leverkusen, Germany) is a selective BET inhibitor that suppresses the expression of MYC and BRD4 proteins and is administered orally.[17] A Phase I dose-escalation study was started in March 2015 but was terminated in 2016 due to toxicity, with DLTs occurring at doses lower than the aimed drug exposure. This trial enrolled eight patients, who were administered oral tablet twice weekly at dose levels of 10 mg/week (*n* = 3), 40 mg/week (*n* = 3), and 80 mg/week (*n* = 2). DLTs were observed in both patients treated at 80 mg, including G3 vomiting, G3 headache, and G2/3 back pain. The most common AEs were nausea, vomiting, headache, back pain, and fatigue. Biomarker analysis indicated a decrease of MYC expression and a linear relationship between dose response and increasing dose. Prolonged disease stability was observed in two patients, but no objective response was achieved in any patient.

## Birabresib (Previously MK-8628/OTX015)

Birabresib (previously MK-8628/OTX015) (Merck Sharp and Dohme Corp., Kenilworth, NJ, USA) is an orally administered BET inhibitor.[18,19] An open-label, Phase Ib, 3 + 3 dose-escalation trial of birabresib to determine DLT and recommended Phase II dose began in October 2014 and enrolled 47 patients (one was not treated) with castration-resistant prostate cancer (CRPC), NMC, and non-small cell lung cancer (NSCLC). In cohort A, birabresib was administered once daily in a fasted state in a 21-day cycle. The starting dose level tested was 80 mg, and the highest dose level tested was 100 mg. In cohort B, birabresib was administered once daily in a fasted state for the first 7 days of a 21-day cycle. The starting dose level was 100 mg and the highest dose level was 160 mg. In cohort A, 24 patients were treated including 19 patients at 80 mg, of which 4 (21.1%) experienced DLTs of G3 thrombocytopenia (*n* = 3) and alanine aminotransferase elevation/hyperbilirubinemia (*n* = 1). Three patients were treated at 100 mg, of which two (66.7%) experienced DLTs of G2 anorexia and nausea (*n* = 1 for each) and G4 thrombocytopenia (*n* = 1). In cohort B, 22 participants were treated, and no DLTs were observed. Among the total 46 participants, 38 experienced AEs, including diarrhea (37%), nausea (37%), anorexia (30.4%), vomiting (26.1%), and thrombocytopenia (21.7%). PR was reported in three patients receiving 80 mg daily (15.8%), all with NMC. The study was completed in March 2017.

A Phase IIa, dose-optimization trial of birabresib began in October 2014 to determine the maximum tolerated dose (MTD) in patients with glioblastoma multiforme (GBM).[20,21] Of the 12 participants, 6 received 80 mg, four received 120 mg, and two received 160 mg in a fasted state once daily throughout a 28-day cycle. All the participants experienced at least one AE. Three participants experienced DLTs including one participant receiving 80 mg daily (G3 thrombocytopenia) and two participants receiving 160 mg daily (G3 thrombocytopenia and G3 hyperbilirubinemia). Other AEs included G1 and G2 diarrhea (33.3%), G2 myalgia (8.3%), and G1 INR increase (8.3%). The best response in all patients was progressive disease. The study concluded that birabresib is safe and well tolerated in patients with GBM, and MTD was declared 120 mg. However, the study was terminated in October 2015 due to limited efficacy.

An open-label, Phase 1b, dose-exploration study to determine the MTD of birabresib in patients with advanced NMC, triple-negative breast cancer (TNBC), and CRPC began in May 2016.[22–24] Thirteen participants were enrolled in cohort A and were to receive 20, 30, or 40 mg of birabresib in a fasted state twice daily for 21 consecutive days per cycle. No patients received more than 20 mg of birabresib. Three participants (23.1%) experienced at least one DLT, including G4 thrombocytopenia, G3 fatigue, and G2 hematuria. Eleven participants (84.6%) experienced a drug-related AE and seven (53.9%) experienced G3 or higher drug-related AEs. The highest efficacy reported was SD in six patients (46.2%), all with CRPC. The study was terminated in April 2017 due to low efficacy and not because of safety reasons.

### BMS986158

BMS986158 (Bristol-Myers Squibb, New York City, NY, USA) is a selective oral BET inhibitor. As of 2018, 69 patients have been enrolled in a dose-escalation trial of BMS986158.[25] BMS986158 was administered at doses of 0.75, 1.25, 2.0, 3.0, and 4.5 mg qd across the following three schedules: schedule A was 5 days on/2 days off (*n* = 31), schedule B was 14 days on/7 days off (*n* = 8), and schedule C was 7 days/14 days off (*n* = 29). Pharmacokinetic (PK) analysis showed T_max_ of 2–4 h, T_1/2_ of 33–82 h, and linear PK. DLT of thrombocytopenia was observed and was reversible. Nearly 63% of the patients experienced AEs, the most common of which were diarrhea (34%), thrombocytopenia (28%), and fatigue (16%). Among four patients with NMC, one patient treated at 2.0 mg on schedule A was treated for 279 days and experienced a 16% tumor reduction. Clinical correlates included gene transcription for biomarkers of BET activity (e.g., CCR2 and HEXIM1) in peripheral blood cells, which were dose dependent and reversible.

### GSK525762

GSK525762 (GlaxoSmithKline, Brentford, UK) is administered as an oral tablet that selectively inhibits expression of the BET protein.[26] A Phase I, open-label, 3 + 3 dose-escalation trial began in 2012. Part I of the trial was completed in 2016 with seventy enrolled patients with NMC and other solid tumor cancers. The starting dose level was 2 mg daily and the highest dose level tested was 100 mg daily. In addition, twice-a-day (BID) schedule was tested, including 20 and 30 mg BID. Preliminary results from part 1 have been reported. PK was dose proportional up to 80 mg qd. DLTs were observed at doses of 60 mg (*n* = 1, 11%), 80 mg (*n* = 3, 14%), and 100 mg (*n* = 1, 11%) during the first 28 days of treatment. The most common AEs at all dose levels were thrombocytopenia (44%), nausea (40%), vomiting (29%), anemia (26%), fatigue (26%), decreased appetite (24%), diarrhea (23%), and dysgeusia (20%). Eleven patients with NMC treated on the once-daily schedule were evaluated for response. Two patients exhibited PR, four patients exhibited SD, and one patient had not yet been evaluated. The recommended dose to use in a Phase II was 80 mg daily. The Phase II expansion is ongoing and will enroll up to 150 participants. No preliminary results have yet been presented.

A separate ongoing Phase Ib trial is studying the effect of GSK525762 with either abiraterone (antiandrogen) (Janssen Pharmaceutica, Beerse, Belgium) or enzalutamide (antiandrogen) (Astellas Pharma, Tokyo, Japan).[27] The study has been active as of 2017 but is no longer recruiting. Thirty-seven patients with CRPC have been enrolled. No preliminary trial results have yet been presented.

### INCB054329

INCB054329 (Incyte Corporation, Wilmington, Delaware, USA) is a potent BET protein inhibitor that is administered orally.[15] As of 2018, the ongoing clinical trial had enrolled 54 patients with solid tumors (*n* = 50) or lymphoma (*n* = 4). INCB054329 was administered at doses of 15–30 mg qd to 15–25 mg bid, on various schedules of 5 days on/2 days off, 4 days on/3 days off, and 7 days on/7 days off. One patient (2%) with NSCLC achieved a PR with 61% decrease. Five patients (10%) had clinical progression, 3 patients (6%) had SD for > 6 months, and 14 patients (28%) had SD for < 6 months. The most common AEs included nausea (24%), fatigue (28%), thrombocytopenia (9%), and decreased appetite (24%). Two fatal nontreatment-related AEs were reported (respiratory failure, *n* = 1 and septic shock, *n* = 1). PK demonstrated a T_1/2_ of ~ 2–3 h, with a median exposure of 8.5 weeks. One DLT of thrombocytopenia was observed at 30 mg BID daily. Twenty milligram BID was the selected dose to be used in future trials.

### INCB057643

INCB057643 (Incyte Corporation, Wilmington, DE) is an orally administered small-molecule BET inhibitor.[15,16,28] An open-label, four-part study to determine the safety and efficacy of INCB057643 in patients with advanced malignancies began in May 2016. Patients enrolled in part 1 of the trial received a starting dose of 8 mg, 12 mg, or 16 mg administered once daily during a 21-day cycle. Part 2 of the study further evaluated the safety, efficacy, PK, and pharmacodynamics (PD) of INCB057643. Patients in part 2 received 12-mg administered once daily during the 21-day cycle. Of the 16 patients enrolled in part 1, one experienced DLT at 16 mg (G3 increased INR), and 12 mg was determined to be the MTD and the recommended dose for part 2. The most common AEs in part 1 were decreased appetite in seven patients (43.8%), nausea in six patients (37.5%), and hyperglycemia in five patients (31.3%). Of the 13 patients enrolled in part 1 with solid tumors, 11 were evaluable for efficacy and one of those patients had SD > 6 months. The most common AE among the 16 patients enrolled in part 2 was nausea in four patients (25%). Of the 14 patients enrolled in part 2 with solid tumors, only two were evaluable for efficacy and both had disease progression. Parts 3 and 4 of the study were designed to evaluate the safety, efficacy, PK, and PD of INCB057643 in combination with standard-of-care agents (gemcitabine, paclitaxel, rucaparib, abiraterone, ruxolitinib, and azacitidine), but the study was terminated in January 2019 for safety reasons and no results have been published.

An open-label, Phase ½ trial with three treatment groups began in January 2017 to evaluate the safety, tolerability, and efficacy of pembrolizumab and epacadostat combined with azacitidine (Group A), INCB057643 (Group B), and INCB059872 (Group C).[19,29] Treatment Group B of the study consisted of two parts. In part one, which followed a 3 + 3 + 3 dose-escalation design, patients orally self-administered INCB057643 qd throughout the 21-day cycle. The patients also received pembrolizumab administered in 30-min intravenous (IV) infusions on the 1^st^ day of each cycle beginning with cycle 2 and epacadostat administered orally twice daily beginning on cycle 2 on day 1. Part 2 of the treatment Group B was a further evaluation of the recommended doses established in part 1, specifically in patients with NSCLC and microsatellite-stable colorectal cancer. No results have been published.

### RO6870810 (previously TEN-010)

RO6870810 (previously TEN-010) (Hoffmann-La Roche, Basel, Switzerland) is a small-molecule BET inhibitor.[30,31] A Phase I, open-label, dose-escalation study was conducted in two parts to determine the safety, PK, tolerability, and efficacy in patients with advanced solid tumors. An estimated total of 84 participants were to be enrolled, including 54 in part A and 30 in part B. Patients enrolled in part A of this study received a starting dose of 0.03 mg/kg of RO6870810 administered by subcutaneous injection, which was escalated to a maximum dose of 0.85 mg/kg. Treatment in part A was administered daily for either 14 days of a 21-day cycle or for 21 days of a 28-day cycle. Part B of the study was designed to further evaluate the safety and efficacy of RO6870810, in which participants received the drug at a dose up to the MTD established in part A. The study began in October 2013 and ended in October 2017, but no results have yet been published.

A Phase Ib study began in November 2017 to evaluate the dose, safety, PK, and efficacy of RO6870810 in combination with atezolizumab at a fixed dose in patients with advanced ovarian cancer and/or TNBC.[17,32] Patients in all the four groups of this study received 1200-mg atezolizumab IV on the 1^st^ day of a 21-day cycle in addition to the dose of RO6870810 designed for each group. In part 1 (dose-escalation group), the participants received doses of RO6870810 administered by subcutaneous injection starting at 0.3 mg/kg (escalated to 0.45 mg/kg and/or 0.65 mg/kg) for the first 14 days of a 21-day cycle. Participants enrolled in part 2 of the study (sequential dose group) received RO6870810 as monotherapy (starting dose 0.3 mg/kg) for the first 14 days of the run-in period, and then received the same dose of RO6870810 qd combined with 1200-mg atezolizumab on the 1^st^ day of every 21-day cycle. The safety, PK, PD, and clinical activity of RO6870810 in patients with TNBC and/or ovarian cancer will be further evaluated in parts 3 and 4 (expansion groups) of the study. No results have yet been published, and the trial has been temporarily suspended for an evaluation of safety and efficacy.

## Discussion

BET inhibitors have entered clinical trials for patients with both hematologic and solid tumor cancers because of promising results in preclinical studies. Seven such BET inhibitors have entered clinical trials for solid tumors. The most common solid tumor cancers treated in these clinical trials include NMC, NSCLC, and CRPC [Table 1].

**Table 1:** Bromodomain and extraterminal protein inhibitor trials

**Drug name**	**Trial phase**	**Tumor type**	**MTD/RP2D**	**Dose-limiting toxicities**	**Terminal half-life**	**Number of patients**	**Antitumor activity**	**Biomarkers examined**
BAY1238097	I	Solid tumor	NA	G3 vomiting, G3 headache, and G2/3 back pain	NA	8	Trial was terminated because DLTs were observed at doses below targeted drug exposure	MYC
BMS986158	I	Solid tumor	NA	Thrombocytopenia	33–82 h	69	¼ patients with NMC, treated at 2.0 mg on schedule A experienced 16% tumor reduction	CCR2 and HEXIM1
Birabresib	Ib	Prostate cancer, NMC, and non-small cell lung cancer	80 mg qd	Thrombocytopenia	NA	47	PR: Three patients with NMC	NA
Birabresib	IIa	Glioblastoma multiforme	120 mg qd	Thrombocytopenia	NA	64	No CR/PR/SD	NA
Birabresib	Ib	NMC, TNBC, and CRPC	20 mg bid	G4 thrombocytopenia, G3 fatigue, and G2 hematuria	NA	13	SD in six patients with CRPC	NA
GSK525762	I	Solid tumor	80 mg qd	Thrombocytopenia and nausea	NA	70	PR: Two patients with NMC SD: Six patients with NMC	IL-6, TNF-α, MCP-1, IL-8
INCB054329	I	Solid tumor and lymphoma	20 mg bid	Thrombocytopenia	2–3 h	54	PR: One patient (2%) with non-small cell lung cancer with 61% decrease of tumor burden	NA
INCB057643	I	Solid tumors	12 mg qd	G3 increased INR	NA	29 Part I :13 Part II: 16	Part I: One of those patients with SD > 6 months Part II: Only two were evaluable for efficacy and both had disease progression	NA
RO6870810 (previously TEN-010)	I	Solid tumors	NA	Not yet reported	NA	Up to 84	Trial recently finished in 2018, no results have been published yet	NA

QD: Once daily, BID: Twice daily, PR: Partial response, SD: Stable disease, CR: Complete response, DLTs: Dose-limiting toxicities, MTD: Maximum tolerated dose, RP2D: Recommended phased II dose, NUT: Nuclear protein in testis, NMC: NUT midline carcinoma, CRPC: Castrate-resistant prostate cancer, INR: International normalized ratio, TNBC: Triple-negative breast cancer, NA: Not available, IL-6: İnterleukin-6, TNF-α: Tumor necrosis factor-alpha

To date, GSK525762, INCB054329, birabresib, and BMS986158 have shown evidence of antitumor activity. Out of the 11 patients with NMC treated with GSK525762, two had a PR and four had SD, and one has not yet been evaluated. Among the four patients with NMC treated with BMS986158, one patient was observed with a 16% tumor decrease. The trial of INCB054329 treated patients with NSCLC, lymphoma, and other solid tumor cancers. One patient achieved a PR with 61% decrease and 17 patients with SD in a cohort of 54 patients (three patients with SD > 6 months and 14 patients with SD < 6 months). Forty-seven patients (one was not treated) with CRPC, NMC, and NSCLC were treated with birabresib. A PR was reported in three patients diagnosed with NMC. In the trial of INCB057643, one patient observed SD for > 6 months. Additional trials are still ongoing, with results not yet reported.

All BET inhibitors that have entered clinical trials to date have been administered as oral agents with the exception of RO6870810, which was a subcutaneous injection. PK properties have been observed to be variable among these agents. For example, the half-life of BMS986158 was observed to be 33–82 h, in contrast to INCB054329 which had a half-life of 2–3 h. Consequently, the dosing schedules of each of these agents have varied. For example, GSK525762 was administered daily, whereas BMS986158 had schedules that varied from 5 days on/2 days off, 14 days on/7 days off, or 7 days/14 days off. The differences in PK properties may be attributed to variable binding strength of each agent.

The most common or dose-limiting toxicities observed with BET inhibitors to date include thrombocytopenia, nausea, diarrhea, and fatigue. Grade 3 and 4 thrombocytopenia was observed in 9%–45% of patients who received BET inhibitors but was also reversible and not cumulative. Nausea was observed in 28%–40% of patients. Diarrhea was documented to affect 33%–37% of patients, and fatigue affected 17%–26% of patients who were administered a BET inhibitor. All AEs were reversible and were typically tolerable with supportive medication.

Given the relatively low response rate among patients treated with BET inhibitors to date, a selective biomarker may be a promising development strategy for this class of medication. One potential biomarker for selecting patients may be MYC amplification or other alterations in the MYC signaling pathway. A preclinical study of 673 cell lines incubated with the BET inhibitor JQ1 showed a strong correlation between MYC amplification and sensitivity to the drug, but of the 99 sensitive cell lines, only four had MYC amplification.[5] The importance of MYC amplification is illustrated by a trend of decreasing MYC expression among responding patients who received treatment with BET inhibitor BAY1238097.[19] However, despite this evidence suggesting a relationship between MYC expression and response to BET inhibition, there is currently no published data to confirm that patients with MYC amplification experience greater clinical benefit. Additional analyses are ongoing.

Data from preclinical studies suggest that BET inhibitors may be effective when administered in combination with epigenetic inhibitors, kinase inhibitors, immune checkpoint inhibitors, cell cycle inhibitors, DNA damage repair agents, and chemotherapy.[14] Some examples of preclinical studies of such combinations showing promising results include combinations with PI3K inhibitors in breast, ovarian, and colorectal cancers; extracellular signal-related kinase inhibitors in ovarian cancer; poly ADP ribose polymerase (PARP) inhibitors in ovarian and breast cancer; and HER2 inhibitors in breast cancer.[3] In preclinical lymphoma models, synergistic activity has been observed in combination with Bruton's tyrosine kinase, BI3K, and BCL2 inhibitors, and there are currently two clinical trials studying the effects of BET inhibitors combined with BCL2 inhibitors in patients with relapsed lymphoma.

Due to the high toxicity and limited long-term efficacy observed to date with BET inhibitors, in addition to the development of resistance mechanisms to this class of drugs, it has become increasingly important to explore other agents which target this pathway, which may be potentially more selective, more potent, and/or less toxic than what has already been investigated. One such medication, AZD5153 (AstraZeneca, Cambridge, UK), is a BRD4 inhibitor that has shown antitumor activity in preclinical models, and began the first-in-human clinical trial in June 2017.[33] Of 28 patients enrolled, seven patients experienced Grade ≥ 3 AEs, which were thrombocytopenia and fatigue (7.1% each), anemia, diarrhea, and decreased platelets (3.6% each).[34] Preliminary results established AZD5153 to be tolerated in patients with relapsed/refractory solid tumors and lymphoma at doses up to 30 mg qd.[35] BRD4 may be a potentially promising target for more selective BET inhibition in future.

In summary, BET inhibitors are a promising class of drug, which have shown early antitumor activity in solid tumor cancers. Additional studies are ongoing to further investigate efficacy in specific tumor types.
